# Supplementary material for: Postoperative trachomatous trichiasis and associated factors among adults who underwent trachomatous trichiasis surgery in Ambassel District, North-East Ethiopia
Source: PLoS One. 2024 May 28;19(5):e0304407. doi: 10.1371/journal.pone.0304407 (PMC11132444; doi:10.1371/journal.pone.0304407)
Supplement: Attachment — Submitted filename: Response to Reviewers (2).docx [file pone.0304407.s002.docx]

**Response to the Reviewers**

Dear editor, thank you for considering our manuscript for publication; we have revised the contents based on the reviewer’s comments as below.

Ref: Submission ID : PONE-D-23-08111
Title: "Postoperative Trachomatous Trichiasis  and Associated Factors Among Adults who underwent TT Surgery in Ambassel District, North-East Ethiopia”.
PLOS ONE."
Reviewers' comments:

Reviewer's Responses to Questions

**Comments to the Author**

1. Is the manuscript technically sound, and do the data support the conclusions?

The manuscript must describe a technically sound piece of scientific research with data that supports the conclusions. Experiments must have been conducted rigorously, with appropriate controls, replication, and sample sizes. The conclusions must be drawn appropriately based on the data presented.

Reviewer #1: Partly

Reviewer #2: Yes

2. Has the statistical analysis been performed appropriately and rigorously?

Reviewer #1: I Don't Know

Reviewer #2: Yes

3. Have the authors made all data underlying the findings in their manuscript fully available?

The [PLOS Data policy](http://www.plosone.org/static/policies.action#sharing) requires authors to make all data underlying the findings described in their manuscript fully available without restriction, with rare exception (please refer to the Data Availability Statement in the manuscript PDF file). The data should be provided as part of the manuscript or its supporting information, or deposited to a public repository. For example, in addition to summary statistics, the data points behind means, medians and variance measures should be available. If there are restrictions on publicly sharing data—e.g. participant privacy or use of data from a third party—those must be specified.

Reviewer #1: Yes

Reviewer #2: Yes

4. Is the manuscript presented in an intelligible fashion and written in standard English?

PLOS ONE does not copyedit accepted manuscripts, so the language in submitted articles must be clear, correct, and unambiguous. Any typographical or grammatical errors should be corrected at revision, so please note any specific errors here.

Reviewer #1: No

Reviewer #2: No

5. Review Comments to the Author

Please use the space provided to explain your answers to the questions above. You may also include additional comments for the author, including concerns about dual publication, research ethics, or publication ethics. (Please upload your review as an attachment if it exceeds 20,000 characters)

Journal Requirements:

When submitting your revision, we need you to address these additional requirements.

1. Please ensure that your manuscript meets PLOS ONE's style requirements, including those for file naming. The PLOS ONE style templates can be found at

<https://journals.plos.org/plosone/s/file?id=wjVg/PLOSOne_formatting_sample_main_body.pdf> and

<https://journals.plos.org/plosone/s/file?id=ba62/PLOSOne_formatting_sample_title_authors_affiliations.pdf>.

*Response: We followed all journals requirements.*

2. We note that your Data Availability Statement is currently as follows: [All relevant data are within the manuscript and its Supporting Information files.]

Please confirm at this time whether or not your submission contains all raw data required to replicate the results of your study. Authors must share the “minimal data set” for their submission. PLOS defines the minimal data set to consist of the data required to replicate all study findings reported in the article, as well as related metadata and methods (<https://journals.plos.org/plosone/s/data-availability#loc-minimal-data-set-definition>).

For example, authors should submit the following data:

- The values behind the means, standard deviations and other measures reported;

- The values used to build graphs;

- The points extracted from images for analysis.

Authors do not need to submit their entire data set if only a portion of the data was used in the reported study.

If your submission does not contain these data, please either upload them as Supporting Information files or deposit them to a stable, public repository and provide us with the relevant URLs, DOIs, or accession numbers. For a list of recommended repositories, please see <https://journals.plos.org/plosone/s/recommended-repositories>.

If there are ethical or legal restrictions on sharing a de-identified data set, please explain them in detail (e.g., data contain potentially sensitive information, data are owned by a third-party organization, etc.) and who has imposed them (e.g., an ethics committee). Please also provide contact information for a data access committee, ethics committee, or other institutional body to which data requests may be sent. If data are owned by a third party, please indicate how others may request data access.

Response: We have uploaded as supportive document files.

3. Your ethics statement should only appear in the Methods section of your manuscript. If your ethics statement is written in any section besides the Methods, please move it to the Methods section and delete it from any other section. Please ensure that your ethics statement is included in your manuscript, as the ethics statement entered into the online submission form will not be published alongside your manuscript.

[Note: HTML markup is below. Please do not edit.]

Response: we moved the ethics statement from declaration section to method section (page #11)

Reviewer #1: This paper describes a study in Ambassel District in Ethiopia where a regression was performed to determine if post-operative TT is correlated with several variables. The results demonstrate a correlation between post-operative TT and patient age, duration since surgery, epilation history, face washing, knowledge about trachoma, and post surgery azithromycin. This information is helpful to the community to inform resource alignment for supporting TT surgery. The study appears to have been designed and performed appropriately. However, the writing in this manuscript is not sufficient for publication at this time. A thorough copy edit is needed and then I would be very happy to re-review.

It is my understanding that this study was focused on trachomatous trichiasis rather than all cause trichiasis. If this assumption is correct, I recommend the following copy edits.
- Reword first paragraph to include background on what TT is and how it’s addressed (trachoma is the world’s leading cause of preventable blindness…morbidity from trachoma is called trachomatous trichiasis (TT)… TT can be managed through surgery … details of what that surgery entails…etc.)
- If you include this information about what trachoma is and where TT comes into play in the natural progression of the disease then you no longer need the paragraph about the WHO grading system.

*Response: Thank you for your feedback. We have revised as the reviewer’s suggestions(lines#55-75).*

- Why is the wealth index included as a figure – this information does not contribute to the study and so does not warrant a figure.

*Response: Thank you for the feedback. We removed figure 1 and its descriptions (page#11, lines 246-247).*

- The details on the status of implementation in Ethiopia should come after the paragraph on the global situation. Additionally, the Ethiopia sentence is unclear and needs a copy edit.

Response: Thank you for the feedback. We revised as your recommendations.

The methods sections read like a protocol rather than a manuscript. Please consolidate the language into one clear Methods section. For example, the sample size section could be one sentence in your methods where you describe the power and confidence of your sample size. Remove variables and operational definitions sections.

Response: We revised it.

The analysis is generally appropriate. However, I’m concerned about confounding among some of the variables. I am also not convinced that martial status, responsibility in the household, and occupation should be included in the analysis as the sample is not balanced for these categories. Marital status is 74% married and 15% widowed (which may be confounded with age). Responsibility in the house was 90% head. Occupation was 93% farmer.

Response: We acknowledge the reviewer’s concern. However, we believe that these potential confounding variables would be managed in the analysis stage; since we conducted multivariable logistic regression model.

The claims in this paper align with previous literature and the authors demonstrate this consistency in the discussion. However, there is need for tightening the writing of this section and drawing the “so what” out a bit more.

Response: Thank you for the feedback. We tried to show the clinical and public health implications the results in the discussion and conclusion sections.

Specific copy edits:
- Please use consistent capitalization in the title of this paper
- Consistently use either trichiasis or trachomatous trichiasis

Response: Thank you for the feedback. We used Trachomatous Trichiasis throughout the document.

- When providing data do not use terms like “nearly” or “about” if the value is 92% then just say that.
- Write out the word “percent” when using it on its own in a sentence rather than using %.

Response: Thank you for the feedback. We followed your suggestions.

- Table 3. Remove “surgeons code” variables – unless you ran an analysis to see if there was a correlation between a particular surgeon and post-operative TT. If you did this, then please include in the text of the manuscript.

*Response: We have analyzed this variable and there was no association between surgeon code and the outcome variable.*

Select more meaningful Key Words

Response: Thank you for your feedback. We revised key words.

Reviewer #2: First and foremost, I'd like to thank the authors of this research for their contribution to Assessing the Postoperative Trichiasis and Associated Factors among Adults who underwent Trachomatous Trichiasis Surgery in Ambassel District, North-East Ethiopia. Herewith my comments and suggestions on each of the manuscript sections
1. Title
Seems good
2. Introduction
• I didn't comprehend the research's novelty. By properly demonstrating why this research was centered on this title, the authors of this manuscript should anticipate to convix both outsiders and insiders of the subjects. In short, the uniqueness of the study should be highlighted.

*Response: thank you for your feedback. We revised the introduction section to address the reviewer’s concerns.*

3. Method section

• The author of this manuscript should also explain how validity and reliability were ensured.

*Response: the validity and reliability of the study was insured through following standard procedures and using pretested data collection tools.*

• How are the effects of confiding variables managed?

*Response: We believe that potential confounding variables would be managed in multivariable logistic regression analysis.*

4. Results
• Good looking!

*Response: thank you for your acknowledgment.*

5. Discussion

• First, the author should clearly interpret what the results mean. Second, compare the findings with comparable populations and settings (attention to the level of heath care facility included). Third, state the possible explanation for the disparity between this study's findings and prior literature after careful review. Fourth, the possible public health implications should also be stated in the discussion section. The author should also state the strengths and limitations of the study.
• Firth, methodological discussion should be recommended (Attention to internal and external validity).

*Response: Thank you for your feedback and suggestions. We revised the whole discussion section to address the reviewer’s concerns.*

6. Conclusions and recommendations.
• The concussions section seems what expected from discussion section. For instances: The prevalence rate of postoperative Trichiasis in the study area was higher than most Ethiopian studies.
• The recommendation seems based on general facts. So, make a sound, actionable, and conclusion-based recommendation after revising the conclusion section.

*Response: We revised conclusion and recommendation sections in line with the objective of the study and our findings.*

7. References
• Should be relevant to the population and setting of this investigation.

Response: Thank you for your feedback.
