# Supplementary material for: Postoperative trachomatous trichiasis and associated factors among adults who underwent trachomatous trichiasis surgery in Ambassel District, North-East Ethiopia
Source: PLoS One. 2024 May 28;19(5):e0304407. doi: 10.1371/journal.pone.0304407 (PMC11132444; doi:10.1371/journal.pone.0304407)
Supplement: Attachment — Submitted filename: Response to Reviewers.docx [file pone.0304407.s003.docx]

**Response to the Reviewers**

Dear editor, thank you for considering our manuscript for publication; we have revised the contents based on the reviewer’s comments as below.

Ref: Submission ID: PONE-D-23-08111R1

Title: Postoperative Trachomatous Trichiasis and Associated Factors among Adults who underwent Trachomatous Trichiasis Surgery in Ambassel District, North-East Ethiopia.

PLOS ONE

Reviewers' comments:

Reviewer's Responses to Questions

Comments to the Author

1. If the authors have adequately addressed your comments raised in a previous round of review and you feel that this manuscript is now acceptable for publication, you may indicate that here to bypass the “Comments to the Author” section, enter your conflict of interest statement in the “Confidential to Editor” section, and submit your "Accept" recommendation.

Reviewer #1: (No Response)

2. Is the manuscript technically sound, and do the data support the conclusions?

The manuscript must describe a technically sound piece of scientific research with data that supports the conclusions. Experiments must have been conducted rigorously, with appropriate controls, replication, and sample sizes. The conclusions must be drawn appropriately based on the data presented.

Reviewer #1: Partly

3. Has the statistical analysis been performed appropriately and rigorously?

Reviewer #1: Yes

4. Have the authors made all data underlying the findings in their manuscript fully available?

The PLOS Data policy requires authors to make all data underlying the findings described in their manuscript fully available without restriction, with rare exception (please refer to the Data Availability Statement in the manuscript PDF file). The data should be provided as part of the manuscript or its supporting information, or deposited to a public repository. For example, in addition to summary statistics, the data points behind means, medians and variance measures should be available. If there are restrictions on publicly sharing data—e.g. participant privacy or use of data from a third party—those must be specified.

Reviewer #1: Yes

5. Is the manuscript presented in an intelligible fashion and written in standard English?

PLOS ONE does not copyedit accepted manuscripts, so the language in submitted articles must be clear, correct, and unambiguous. Any typographical or grammatical errors should be corrected at revision, so please note any specific errors here.

Reviewer #1: No

6. Review Comments to the Author

Please use the space provided to explain your answers to the questions above. You may also include additional comments for the author, including concerns about dual publication, research ethics, or publication ethics. (Please upload your review as an attachment if it exceeds 20,000 characters)

Reviewer #1: I appreciate the author’s hard work on this study and believe it’s important for this information to be available for the global community. However, the paper still requires significant copy-editing. I have provided some initial suggested edits below, but the full paper would greatly benefit from a full copy edit by someone with experience publishing in an English language journal.

*Response: We thank you the reviewer’s constructive comments and suggestions. We copy edited the whole manuscript for typo and grammar errors.*

Since you abbreviate trachomatous trichiasis as TT early in the paper, continue using TT throughout rather than spelling out the full name.

Same comment on using POTT throughout.

*Response: Thank you for your suggestions. We corrected throughout the document.*

Line 56 – rather than stating “…transmitted through contact with eye discharge” I recommend a broader statement, such as “…transmitted through contact with an infected person”. This is because the bacteria could be transmitted via nasal discharge or other fomites. (Trachoma - StatPearls - NCBI Bookshelf (nih.gov))

*Response: We corrected it, (line 56).*

Line 58 – change “resulting in excruciating pain….” To say something like “causing pain and overtime can result in scarring of the cornea”. The large spectrum of clinical manifestations of TT means that some people do not develop scarring, but many do. So better for this language to reflect this. You all should cite this statement.

*Response: Thank you for your suggestions. We edited from the document (line 58).*

Line 59 – same comment at line 58 about the large spectrum of clinical manifestations. I would say “If left unmanaged TT can lead to visual impairment and blindness.” You all should cite this statement.

*Response: We acknowledge the reviewer’s effort and we edited (line 59).*

Line 60 – Trachoma is the leading cause of preventable blindness. I don’t think this is the correct citation. Please double check.

*Response: We acknowledge the reviewers concern. We found that this statement is duplicated in the lines 78-80, hence, we merged it and the correct citation is reference no.7 (line 80).*

Line 61 – TT can be managed through surgery which involves….(briefly describe the procedure and cite the WHO manual)

*Response: We included the types of TT surgery procedures (lines 63-65).*

Line 68 – what is “the plan”? since you give a true value “173,945” remove “about”. I think what you are saying is “In Ethiopia, 173,945 received TT surgery and 39,339,311 people received antibiotics through mass drug administration in 2017.”

*Response: Thank you. We revised based on the reviewer’s suggestions (line 68).*

Line 71 – all villages received health education….

*Response: We edited it (line 76).*

Line 75/76 – suggest rewording “TT creates economic loss due to reduced productivity resulting form blindness and visual impairment.”

*Response: We edited the manuscript based on the reviewer’s suggestions (lines 80-82)*

Line 77 – POTT negatively impacts perception and uptake of surgery in communities. Individuals are less likely to seek surgery when they witness POTT in their community. Additionally, re-operation has…..”

*Response: We thank you the reviewer’s effort. We edited the statements (lines 83-88).*

Line 85 – POTT

*Response: Corrected.*

Line 88/89 – confusing wording

*Response: We reworded the statements (lines95-99).*

Line 219 – first time to mention PLTR – I suggest mentioning this approach in the background and you also need to spell out the acronym the first time you use it.

*Response: The PLTR has been introduced in the background section of the manuscript (line 65).*

Aside from copy-edit. Could the authors please explain how they accounted for quality of original surgery? Were all the surgeons in the study of a certain caliber?

*Response: The surgeons were Integrated Eye Care Workers who have had a special training on TT surgery. Except the potential intrinsic individual quality differences they took similar training.*
