# Supplementary material for: Postoperative trachomatous trichiasis and associated factors among adults who underwent trachomatous trichiasis surgery in Ambassel District, North-East Ethiopia
Source: PLoS One. 2024 May 28;19(5):e0304407. doi: 10.1371/journal.pone.0304407 (PMC11132444; doi:10.1371/journal.pone.0304407)
Supplement: Attachment — Submitted filename: Response to Reviewers.docx [file pone.0304407.s004.docx]

**Response to the Reviewers**

Dear editor, thank you for considering our manuscript for publication; we have revised the contents based on the reviewer’s comments as below.

Ref: Submission ID: [PONE-D-23-08111R2] - [EMID:07797ed50f9ecbe2] .

Title: Postoperative Trachomatous Trichiasis and Associated Factors among Adults who underwent Trachomatous Trichiasis Surgery in Ambassel District, North-East Ethiopia.

PLOS ONE

Additional Editor Comments:

Dear authors,

Both reviewers suggest the acceptance of the manuscript. Below find some more comments to improving the document.

1. In the abstract and other sections, put the AOR values within the brackets.

*Response: Revised as the editors suggestions (lines 37-43).*

2. Improve the writing style throughout the document. For instance, the statement “A multi-stage sampling technique was used to employ a total of 506 individuals” could be rephrased.

*Response: We revised the whole document for formatting and typo errors.*

3. Carefully check typo and punctuation errors throughout. e.g., infections off….. infections of; In the Amhara region 91,977 persons…..In the Amhara region, 91,977 persons

Response: *We revised the whole document for formatting and typo errors.*

4. The 4th paragraph of the introduction needs to be moved up (either integrated into the first paragraph or so). You cannot talk about Ethiopia and Amhara region before global reports. I generally recommend revising the flow of information in the introduction section.

*Response: Thank you for your comment. We moved up 4^th^ paragraph to the 3^rd^ paragraph place (lines 68-71).*

5. Justification needs to be supported with references. E.g., “Previous studies done in Ethiopia were few and limited only in urban areas” include these studies as references.

*Response: we included references14 and 15.*

6. The authors applied correction formula, which was not actually required.

*Response: Correction formula was applied as a rule of thumb since the source population was small, that is 2133.*

7. Sampling following house number does not seem acceptable as there are scattered households without numbering.

*Response: In this time, there is the so called community health information system (CHIS) or family folder run in the Ethiopian health extension program; in which each household assigned a specific identification number. Hence, study participants were tracked through this system.*

8. In the tables, add the SI units for some variables.

*Response: we revised as the editors suggestions.*

9. I also recommend operational definitions for some variables. e.g., knowledge, type of latrine, toilet utilization.

*Response: We have operationalized some variables as the editor recommended (lines 118-122).*

Reviewers' comments:

Reviewer #2: Postoperative Trachomatous Trichiasis and Associated Factors Among Adults who underwent Trachomatous Trichiasis Surgery in Ambassel District, North-East Ethiopia.

Authors have incorporated all the correction suggested by reviewer. Now it may be accepted

*Response: Thank you for your considerations.*

Reviewer #3: The paper entitled ‘Postoperative Trachomatous Trichiasis and Associated Factors Among Adults who underwent Trachomatous Trichiasis Surgery in Ambassel District, North-East Ethiopia’ The title is very important and it is tries to address the current hot topics and the study makes significant contributions to the study area and beyond.

10. Line 75: ‘POTT negatively impacts perception and uptake of surgery in communities.’ A sentence cannot start by an abbreviation/acronym, so please write the long form of ‘POTT’.

*Response: Revised as the reviewer’s suggestion (line, 75).*

11. Lines 145 &146: Include the Institutional Review Board (IRB) Committee decision reference number

*Response: We included the IRB committee protocol number (line 146).*

12. Line 172: Editorial issue; ‘The Prevalence of Postoperative Trachomatous Trichiasis’. Here ‘Trachomatous’ should be written in bold.

*Response: Thank you for your reminder: we have edited it (line 172).*

13. Line 175: ‘Factors associated with postoperative Trachomatous Trichiasis’. Here also ‘Trachomatous’ should be written in bold.

*Response: Thank you for your reminder: we have edited it (line 175).*
